# WASP family proteins, more than Arp2/3 activators

**DOI:** 10.1042/BST20160176

**Published:** 2016-10-19

**Authors:** Joe J. Tyler, Ellen G. Allwood, Kathryn R. Ayscough

**Affiliations:** Department of Biomedical Science, Firth Court, University of Sheffield, Sheffield S10 2TN, U.K.

**Keywords:** actin, Arp2/3, Las17, nucleation, polyproline, WH2

## Abstract

Wiskott–Aldrich syndrome protein (WASP) family proteins have been extensively characterized as factors that promote the nucleation of actin through the activation of the protein complex Arp2/3. While yeast mostly have a single member of the family, mammalian cells have at least six different members, often with multiple isoforms. Members of the family are characterized by a common structure. Their N-termini are varied and are considered to confer spatial and temporal regulation of Arp2/3-activating activity, whereas their C-terminal half contains a polyproline-rich region, one or more WASP homology-2 (WH2) actin-binding domains and motifs that bind directly to Arp2/3. Recent studies, however, indicate that the yeast WASP homologue Las17 is able to nucleate actin independently of Arp2/3 through the function of novel G-actin-binding activities in its polyproline region. This allows Las17 to generate the mother filaments that are needed for subsequent Arp2/3 recruitment and activation during the actin polymerization that drives endocytic invagination in yeast. In this review, we consider how motifs within the polyproline region of Las17 support nucleation of actin filaments, and whether similar mechanisms might exist among other family members.

## Background

A fundamental understanding of how a cell responds to its environment, in order to drive changes in cell physiology, is critical if we are to make relevant and appropriate interventions in the context of disease states. Many years of research have demonstrated that the actin cytoskeleton is a focal point of regulation; however, there are still large gaps in our understanding of mechanisms governing *de novo* actin filament formation and regulation in the context of membranes.

**Figure 1.**
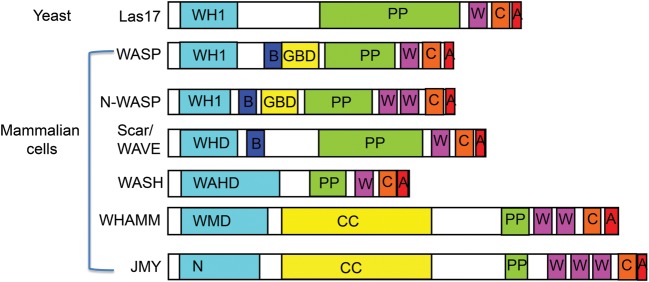
Schematic diagram showing domains of WASP family proteins. N-terminal domains depicted are in pale blue — WASP homology 1 (WH1); WAVE homology domain (WHD); WASH homology domain (WAHD) and the WHAMM membrane interaction domain (WM) or N-terminal domain (N). Other domains are: B, basic region; GBD, GTPase-binding domain; CC, coiled coil region; P, proline-rich domain; W, the WASP homology-2 (WH2) domain; C, the connecting or central domain; A, acidic domain. W, C and A together are referred to as the WCA domain. In general, the N-terminal domains are thought to confer spatiotemporal regulation on the protein, while the WCA regions at the C-terminal are required for activation of Arp2/3.

The initial stage of filament formation, the assembly of a nucleus of 3–4 actin monomers, is energetically unfavourable, highly concentration dependent. In the absence of nucleation, promotion factors occur only slowly. However, following nucleation, growth of the polymer proceeds rapidly [1–3]. There are two well-characterized actin nucleation machineries in eukaryotes. Arp2/3, which is a seven-subunit complex, proposed to generate a branched cortical actin network in cells, and formins that generate unbranched filaments that are often bundled together [1,2]. The Arp2/3 complex does not nucleate actin *de novo* and requires both a nucleation promotion factor (NPF) and an existing filament from which to form a branch [4]. While many proteins have been described to act as NPFs for Arp2/3, the most studied group of these proteins is the Wiskott–Aldrich syndrome protein (WASP) family [5]. Members of this protein family are found across eukaryotic organisms and in all cases are considered to function with Arp2/3 to nucleate actin filaments [6]. The critical part of these proteins to activate Arp2/3 is the C-terminal region, whereas the N-terminal parts are relatively diverse and considered to facilitate spatio-temporal regulation of the individual family members [6]. With their multidomain protein structures, the WASP family of proteins is ideally built for integrating diverse upstream signals to drive appropriate changes in the actin cytoskeleton [6–8]. Figure 1 illustrates many best-characterized mammalian WASP family members and the yeast homologue Las17.

Based on evidence from many studies, it is clear that Arp2/3's function is closely linked with an ability to bind to the C-terminal acidic region of WASP family proteins and to ‘receive’ a monomer bound by the actin-binding motif, WASP homology-2 (WH2). This monomer is proposed to combine with the actin-related moieties, Arp2 and Arp3, to mimic a trimer, which can then act as a nucleus for polymerization, as Arp2/3 is effectively mimicking an actin dimer that then combines with a further monomer delivered by WASP to generate the nucleating trimer [9,10]. This nucleus can then undergo rapid actin polymerization. Indeed, this model of monomer delivery to Arp2/3 to create an actin nucleus is at the heart of our current mechanistic understanding of Arp2/3-based actin nucleation.

A question that remains, however, is the nature of the filaments that Arp2/3 must bind in order to generate the ‘branch’ of polymerized actin. The nature of these ‘mother’ or nascent’ filaments is only just beginning to be addressed. One suggestion is that Arp2/3 will bind to existing actin filaments that are found in the dense cortical actin mesh in many mammalian cells. One limitation of this model is that some membrane sites where actin is known to be nucleated, such as endocytic sites in yeast and endosomes in mammalian cells, do not appear to be surrounded by actin meshworks, so the origin of pre-existing filaments for Arp2/3 recruitment is not clear. A proposal from work in the fission yeast is that the actin filament-depolymerizing and -severing protein cofilin can function to generate short filaments and that these can be recruited by the membrane-tethered protein End4/Sla2 at endocytic sites [11]. Currently, it is not known how the filaments remain assembled during this movement and capture mechanism.

Many pieces of evidence accumulated over the last decade have begun to point to the importance of the polyproline-rich (PPR) region of WASP proteins, not only just as a scaffold region for binding SH3 domain proteins but also as a domain capable of functioning in the regulation of actin dynamics in its own right. One of the earlier pieces of evidence derives from a set of studies comparing the effect of WCA domains from WASP and Scar/Wave with constructs carrying the PPR + WCA and the full-length proteins in the presence of Arp2/3 (see Figure 1 for domain nomenclature). In both cases, the addition of PPR to WCA reduced the lag phase, as defined by the time taken before an increase in pyrene fluorescence (indicative of filament formation) could be detected [12]. Importantly, this enhancement of filament elongation can be viewed as an activity inherent to the PPR region as additional proteins were not used in these assays. Work from the Goode lab added further evidence through an analysis of yeast Las17. In the present study, the effect of full-length Las17 or WCA alone on actin polymerization was compared and, somewhat surprisingly, only the full-length protein nucleated actin in the presence of Arp2/3, while the WCA domain contributed to an increase in filament elongation [13]. Three more recent studies described below have contributed to a greater understanding of the PPR region in the yeast WASP homologue and underpin the importance of this region in the function of this key actin regulator.

## Arp2/3-independent actin nucleation by yeast Las17/WASP polyproline region

Endocytosis in budding yeast requires actin to be polymerized at specific plasma membrane sites. Force generated through actin polymerization, and by bundling actin filaments together, is necessary to drive the required inward membrane invagination against the outward pressure due to cell turgor [14]. A similar requirement for actin during endocytosis is found in mammalian cells when the plasma membrane is under tension [15]. Las17 is recruited to endocytic sites following assembly of various coat complexes and cargo recruitment. Once recruited, there is a highly reproducible sequence of assembly and disassembly events at the sites driving membrane invagination and culminating in scission of a vesicle [16,17]. Unlike mammalian cells, there is not an underlying cortex of actin and it is considered that actin filaments is must be generated *de novo* at new sites of endocytosis.

Las17 is the primary activator of Arp2/3 at yeast endocytic sites [18]. As shown in Figure 1, Las17 has a domain structure broadly similar to those of other WASP family proteins. In particular, it has a C-terminal WCA domain comprising a G-actin-binding WH2 domain and a connecting + acidic (CA) domain that interacts with Arp2/3. In this way, it can supply monomeric actin to filaments nucleated by Arp2/3. It was therefore a surprise when deletion of just the acidic domain or of the entire WCA domain of Las17 caused only rather mild phenotypes in growth and endocytosis [19,20]. This was in contrast with the complete loss of Las17 that caused a severe growth defect, and endocytosis was barely detectable [21]. These experiments suggested that other regions of Las17 were critical for its cellular function. The *in vitro* work from the Goode lab (above) had also indicated that regions upstream from the WCA region were important for an actin nucleation event.

Investigations by our laboratory then revealed that the polyproline region itself is able to bind to actin and that, in fact, this region alone is able to nucleate actin filaments *de novo* in the absence of Arp2/3 [22]. This was unexpected as the region contained no similarity to known actin-binding regions, rather there were many tracts of five proline residues. Using a combination of *in vitro* and *in vivo* assays, the importance of specific proline residues was demonstrated and a model was proposed suggesting that the proline region contained multiple weak sites of actin binding [22]. This property would serve to increase the local concentration of actin, thus promoting conditions for nucleation. Thus, while the nucleation was not as strong as observed with Arp2/3, it could serve to generate *de novo* filaments which themselves could drive recruitment of Arp2/3 for a more substantial burst of nucleation to drive the invagination of membrane.

More recently, work from Feliciano et al. [23] demonstrated the importance of arginine residue pairs (RR349,350; RR382,383) in Las17 that facilitated G-actin binding within the PPR region. Their study focussed on the role of these residues in the context of Arp2/3 and clearly highlighted the importance of the RR pairs for G-actin binding and for rapid polymerization by Arp2/3.

**Figure 2.**
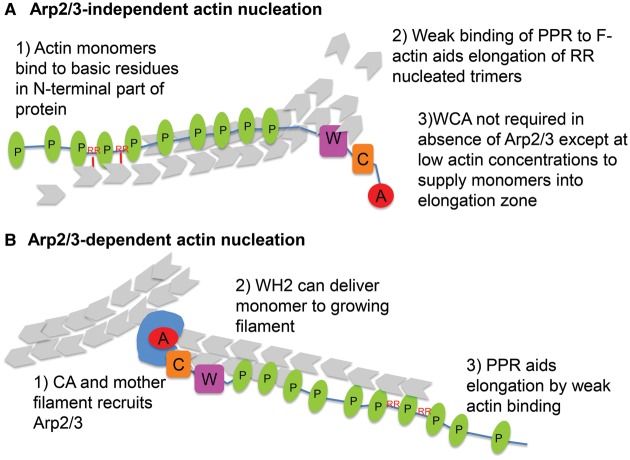
Model showing possible involvement of the PPR of Las17 in actin polymerization. (**A**) In the absence of Arp2/3, Las17 binds and nucleates actin via regions of the PPR region containing paired basic residues. The remainder of the PPR lies alongside the newly formed filament and promotes elongation of the filament via weak actin binding through the repeated proline tracts. The Las17 WH2 has a high affinity for actin monomers and can increase their local concentration when this would otherwise become limiting for PPR-mediated filament elongation. (**B**) In the presence of Arp2/3, a Las17–Arp2/3 complex is formed which binds to a mother filament and initiates branch formation. Branch elongation is enhanced by the PPR domain. W, WH2 domain; C, central or connecting domain; A, acidic domain; P, polyproline repeat motif. Actin is shown in grey and Arp2/3 is shown in blue.

The importance of the polyproline region of Las17 for its primary function was also demonstrated in a study from Lewellyn et al. [24]. In the present study, chimeric proteins were made between Las17 and one of the yeast myosin 1 proteins that also function in the endocytic process. Intriguingly, the critical domains required to support endocytic invagination were the myosin motor domain, a membrane-binding domain (in this case, TH1 from the myosin) and the polyproline region (residues 324–426). Again, the present study shifts the identity for the essential Las17 functions away from the WCA domain onto the polyproline region and would therefore potentially indicate that Arp2/3 activation is a secondary or redundant function of Las17 in the endocytic process. Given that three other proteins at endocytic sites bind to both Las17 and Arp2/3 (Myo3, Myo5 and Abp1), once actin filaments are generated, there are multiple other proteins present that can fulfil the role of Arp2/3 recruitment [19,20]. A diagram outlining our model for the nucleation and elongation of filaments *de novo* by Las17 is depicted in Figure 2.

## Arp2/3-independent actin nucleation by other WASP family proteins

Increased local concentration of actin monomers is known to favour filament nucleation. In Las17, we propose that binding of monomers by the PPR domain allows nucleation to proceed in the absence of Arp2/3. A significant question then is whether other WASP family proteins can use similar mechanisms to also generate filaments *de novo*.

It is notable that in contrast with *Saccharomyces cerevisiae* that has only one WASP family protein with multiple actin-binding sites, mammalian cells have several different WASP family proteins, many of which are expressed in different tissues or active on different membrane compartments [6,25]. As such, they may be adapted to their different situations and have lost functions that are not required in their specific role. However, given that the large majority of WASP family proteins are considered to activate Arp2/3, and that Arp2/3 nucleation activity requires it to bind a mother filament, there is always a need for mother filaments to be generated, and the ability of WASP family proteins to generate such filaments would facilitate close coupling of these two activities.

Four clear routes can be readily identified for WASP family proteins to increase local actin monomer concentration: (1) tandem WH2 domains as in N-WASP and JMY; (2) dimerization as detected for N-WASP; (3) binding of other G-actin-binding proteins such as WIP or profilin and (4) paired basic residue actin binding as in Las17.

JMY is found in the nucleus of numerous cell types, but in more motile cells it is often found at the leading edge [26,27]. More recently, JMY and the related protein WHAMM have both been found to associate with the autophagosome. At this site, WHAMM has been demonstrated to participate in autophagosome biogenesis [28], whereas JMY has been proposed to link actin nucleation to autophagosome maturation [29]. Similar to Las17, JMY can both activate the Arp2/3 complex and nucleate actin filament formation *de novo* [26,27]*.* In the absence of Arp2/3, actin nucleation can be facilitated via three tandem WH2 domains in a similar manner to that observed for the protein Spire, presumably by increasing local actin monomer concentration [30–33].

Multimerization has been considered to be relevant for WASP family protein involvement in nucleation from experiments that demonstrated that GST-tagged WCA domains, which dimerize through their GST moieties, have higher activity in polymerization assays with actin and Arp2/3 than their His-tagged counterparts [34]. One route for dimerization is through the presence of more than one binding site for WCA domains on the Arp2/3 complex, though clearly this route is synonymous with Arp2/3 activation [35,36]. A second route for multimerization, however, relies on the presence of the PPR domain and is facilitated through binding of the WASP family protein by a binding partner with multiple SH3 domains. Such a mechanism has been detected with Nck binding to N-WASP [34]. Again, these routes lead to the presence of multiple WH2 domains in a highly localized region and can therefore contribute to increased local concentration of G-actin. Different conformational orientations of WH2 domains facilitated by intramolecular interactions or multimerization may allow effective regulation of nucleation.

In addition to binding of proteins to bring about multimerization, binding partners often carry additional actin-binding sites. The best characterized of these proteins is WIP (verprolin in *S. cerevisiae*), which binds to the N-terminal region of several WASP family proteins and itself has a WH2 domain as well as PPR regions [37–39]. Furthermore, several groups of proteins bind to the PPR region and may directly or indirectly influence the function of this region with regard to actin dynamics. These include proteins with SH3 domains (binding PXXP motifs), EVH domains (binding FPPPP in VASP) and WW domains (binding PPXY) [40–42]. As well as Nck mentioned above which facilitates N-WASP dimerization, many of these WASP family-binding proteins also bind to F-actin or other actin regulatory proteins. Another protein that has an impact on the activity of some WASP family proteins through binding in the PPR region is the G-actin-binding protein profilin. In the case of Las17, neither we, nor others, have observed an impact of profilin on Las17 function and we consider it most likely that this could be due to the fact that the longest proline tract in the Las17 PPR region is only five residues. Tracts of eight or more prolines, which are found in several WASP family proteins, are considered more likely to bind profilin, whereas for those with an intermediate length of proline tract this aspect of activation is relatively unclear, especially within a cellular context [43–45].

In addition to yeast Las17, the *Drosophila* protein WHAMY is a WASP family protein capable of nucleating actin filaments independently of the Arp2/3 complex [46]. It differs from Las17 and JMY, however, in that it has lost the ability to activate Arp2/3 at all. Instead, it appears to work in tandem with WASP throughout many stages of *Drosophila* development [46]. The interaction of WHAMY and WASP enhances both the Arp2/3-independent activity of WHAMY and the Arp2/3-dependent activity of WASP. Intriguingly, like Las17, WHAMY contains several basic residue pairs within its PPR domain, raising the possibility that its nucleating function might involve a similar mechanism to that we have suggested for Las17. Finally, WASP itself has been shown to contain a very weak Arp2/3-independent actin-nucleating activity within its PPR region [22]. This protein contains a single RR pair just upstream of one of its proline tracts, though the importance of these residues has not yet been reported.

Emerging from these wide-ranging observations is the idea that many WASP family proteins are likely to have functionality, either inherent or through interaction with other proteins, to support *de novo* actin nucleation independently of Arp2/3. However, these activities in general appear to be functionally coupled to the subsequent recruitment and activation of Arp2/3 itself. Important questions for the future will focus on understanding the role of the PPR domain as a region to either facilitate nucleation or elongation of actin filaments; the regulatory links between Arp2/3-independent and -dependent nucleation processes; the extent of nucleation required to generate mother filaments within specific cell settings for Arp2/3 recruitment and defining the conformational arrangements of monomers bound to WASP family proteins to determine how distinct organizations favour nucleation or sequestration of actin.

## Abbreviations

CA, connecting + acidic; EVH, enabled VASP homology; NPF, nucleation promotion factor; PPR, polyproline-rich; WH2, WASP homology-2; WASP, Wiskott–Aldrich syndrome protein.

## Funding

J.J.T. is supported by a White Rose Biotechnology and Biological Sciences Research Council (BBSRC) studentship [BB/J014443/1] and E.G.A. is supported by a BSBRC project grant [BB/N007581/1] to K.R.A.
